# The Reversible Neurotoxic Effects of Methylmercury on the Dorsal Root Ganglion: Temporal Dynamics in Rats

**DOI:** 10.3390/ijms27010116

**Published:** 2025-12-22

**Authors:** Yo Shinoda, Kaito Yamashiro, Ayaka Matsuki, Yuka Sekiguchi, Satoshi Tatsumi, Shino Homma-Takeda, Oki Sekizawa, Marika Abe, Misaki Ozawa, Eiko Yoshida, Yasuhiro Shinkai, Tsutomu Takahashi, Yasuyuki Fujiwara, Toshiyuki Kaji

**Affiliations:** 1 Department of Environmental Health, School of Pharmacy, Tokyo University of Pharmacy and Life Sciences, 1432-1 Horinouchi, Hachioji 192-0392, Tokyo, Japan; kitymsr@toyaku.ac.jp (K.Y.); tsutomu@toyaku.ac.jp (T.T.); yasuyuki@toyaku.ac.jp (Y.F.); 2 Institute for Radiological Science, National Institutes for Quantum Science and Technology, 4-9-1 Anagawa, Inage, Chiba 263-8555, Chiba, Japan; takeda.shino@qst.go.jp; 3 Japan Synchrotron Radiation Research Institute, 1-1-1 Kouto, Sayo 679-5198, Hyogo, Japan; sekizawa@spring8.or.jp; 4 Global Science Campus, Japan Science and Technology Agency (JST), 4-1-8 Honcho, Kawaguchi 332-0012, Saitama, Japan; 5 Shibuya Junior & Senior High School, Shibuya Kyoiku Gakuen, 1-21-18 Shibuya, Shibuya 150-0002, Tokyo, Japan; 6 Hiroo Gakuen Senior High School, 5-1-14 Minamiazabu, Minato 106-0047, Tokyo, Japan; 7 Sustainable System Research Laboratory, Central Research Institute of Electric Power Industry, 1646 Abiko, Abiko 270-1194, Chiba, Japan; yoshida3881@criepi.denken.or.jp; 8 Faculty of Pharmaceutical Sciences, Tokyo University of Science, 6-3-1 Niijuku, Katsushika 125-8585, Tokyo, Japan; 9 Environmental Biology Laboratory, School of Life Sciences, Tokyo University of Pharmacy and Life Sciences, 1432-1 Horinouchi, Hachioji 192-0392, Tokyo, Japan; yshinkai@toyaku.ac.jp

**Keywords:** methylmercury, dorsal root ganglion, neurotoxicity, immunohistochemistry, sensory neuron, recovery

## Abstract

Methylmercury (MeHg) is a well-known environmental neurotoxicant that preferentially affects sensory neurons in the peripheral nervous system. While sensory-dominant neuropathy has long been described in Minamata disease, the temporal dynamics of dorsal root ganglion (DRG) injury and recovery remain incompletely understood. In this study, Wistar rats were exposed to MeHg for five consecutive days, followed by a two-day treatment-free period; this regimen was repeated once. The DRG and peripheral sensory fibers were analyzed up to 70 days after exposure. Histological and immunohistochemical analyses, DNA microarrays, and mercury quantification and distribution mapping were performed. The A-fiber density was significantly reduced at Day 14 but recovered by Day 70, whereas C-fibers showed no significant change. The total number of DRG neurons remained stable. Immunohistochemical analyses demonstrated that subtype marker-selected neurons (NF, TrkA, FAM19A1, TAC1, SST) decreased at Day 14 and gradually recovered thereafter. DNA microarray analysis at Day 14 revealed a broad downregulation of DRG neuronal subtype marker genes. The mercury concentration in the DRG peaked at Day 14 and declined to the control level by Day 70, with in situ imaging confirming preferential accumulation in DRG neurons. These data suggest that the short-term MeHg exposure caused a transient functional suppression of DRG neurons without widespread neuronal loss. The selective and reversible downregulation of neuronal phenotypes, coupled with preferential Hg accumulation in DRG neurons, underlies the sensory-dominant and potentially reversible features of MeHg neurotoxicity.

## 1. Introduction

Methylmercury (MeHg) poisoning, most notably recognized in Minamata disease, is characterized by predominant sensory impairments [1,2,3]. Sensory disturbances primarily affecting the distal extremities have been well documented in many patients with Minamata disease, and classical neuropathological studies demonstrated the degeneration of sensory fibers and dorsal root ganglion (DRG) neurons as key substrates of this clinical phenotype [4,5,6,7]. However, the temporal aspects of DRG injury and the capacity for recovery after MeHg exposure remain poorly understood.

In our previous study, we observed several pathological alterations in the DRG of rats exposed to MeHg, including a reduction in NeuN-positive neuronal cells, loss of sensory nerve fibers, accumulation of macrophages, and proliferation of Schwann cells [8]. These findings were obtained during the acute phase of the MeHg exposure (Day 14 after exposure onset) and demonstrated marked damage to the DRG and associated sensory nerve fibers, accompanied by various histopathological changes. In contrast, a long-term assessment of sensory dysfunction in similar MeHg-exposed rats revealed a transient attenuation of pain-specific sensitivity during the acute phase, followed by a gradual recovery over time [9]. However, the histological changes occurring in the DRG and sensory nerve fibers during the transition from the acute phase to recovery remain unclear. Previous in vitro studies have shown that DRG neurons can undergo cell death, such as apoptosis or necroptosis, mediated by tumor necrosis factor-α (TNF-α) [10]. Nevertheless, the corresponding cellular and molecular events that occur in vivo following MeHg exposure have not yet been elucidated.

The DRG is an organ that contains peripheral sensory neurons responsible for receiving various types of sensory information from both inside and outside the body—including touch, pressure, vibration, proprioception, temperature, itch, and chemical stimuli—and transmitting them to the central nervous system [11,12,13]. The histological architecture of the DRG consists of neurons of widely varying sizes—albeit generally larger than most other cell types—tightly enveloped by satellite glial cells (SGCs), along with Schwann cells, vascular endothelial cells, and other associated cellular populations [14,15,16]. It has become increasingly evident that DRG neurons exhibit remarkable heterogeneity not only in the sensory modalities they encode and in their morphological characteristics but also at the molecular level, as reflected by their highly diverse gene expression profiles [17,18,19]. In addition, the DRG is uniquely susceptible to toxicants due to its high metabolic activity, specialized ion channel expression, and limited blood–nerve barrier [14,20,21,22]. In fact, chemotherapy-induced peripheral neuropathy (CIPN) targets the DRG as its primary organ, a phenomenon thought to stem from the aforementioned characteristics [23,24]. In addition to the toxicant sensitivity of the DRG, recent studies indicate that MeHg preferentially accumulates in DRG neurons, compared with motor neurons, through transport systems such as L-type amino acid transporter 1 (LAT1) [25]. Yet, whether this accumulation causes permanent cell loss or reversible phenotypic suppression remains unresolved.

In this study, we exposed Wistar rats to MeHg for 12 days with 2 non-exposed days on the 6th and 7th days and examined the DRG and peripheral sensory fibers over 70 days. Using immunohistochemistry, DNA microarrays, and mercury mapping, we sought to clarify the dynamics of neuronal injury and recovery in the DRG.

## 2. Results

### 2.1. Temporal Dynamics of Peripheral Sensory Fibers

**Figure 1:**
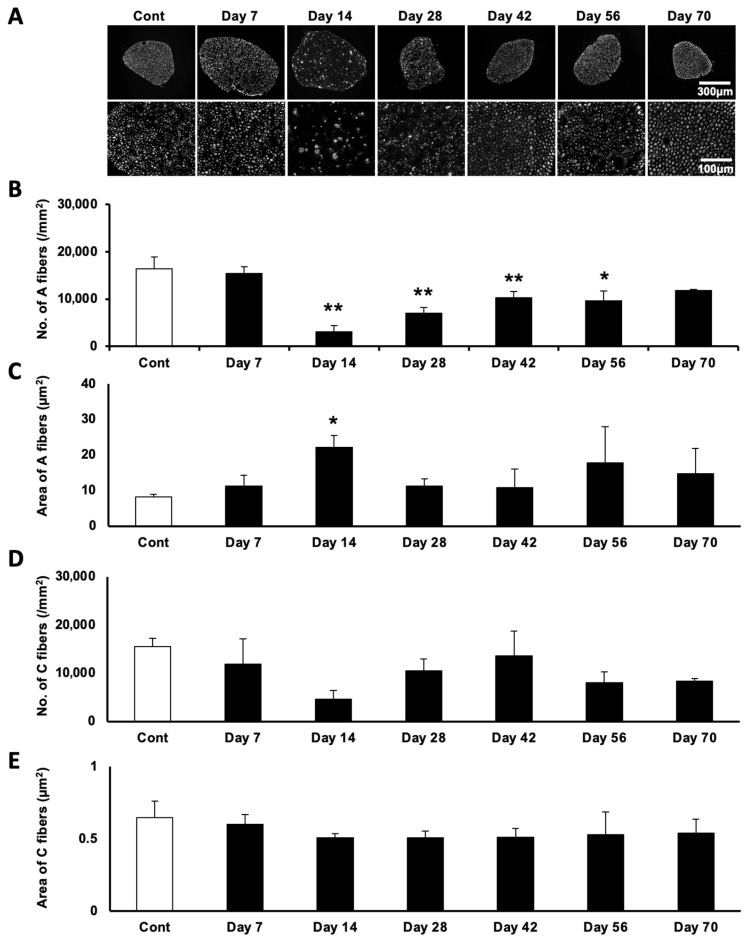
Degeneration and subsequent recovery of sensory axon in MeHg-exposed rat. (**A**) Representative images of sensory axon fibers immunolabeled by NF. Total (upper) and magnified (lower) view in each time point. The scale bars apply to all corresponding images. (**B**) The average number (*p* = 1.85 × 10^−6^, F = 21.4) and (**C**) area (*p* = 0.033, F = 3.11) of A-fibers. (**D**) The average number (*p* = 0.053, F = 2.71) and (**E**) area (*p* = 0.408, F = 1.11) of C-fibers. Data are shown as mean ± SD (*n* = 3). One-way ANOVA with post hoc Tukey–Kramer test. The degrees of freedom were 20 (total), 6 (between groups), and 14 (error variance) for all analyses. The corresponding *p*-values and F-values are shown in each figure legend. * *p* < 0.05 and ** *p* < 0.01 compared with control, respectively.

Sensory fibers are known to be markedly affected in patients with Minamata disease. We therefore conducted a quantitative analysis of both the number and the thickness of sensory fibers in MeHg-exposed rats (Figure 1). Based on the fiber diameter, sensory fibers were classified into two categories: A-fibers (≥1.5 µm) and C-fibers (<1.5 µm). The number of A-fibers was significantly reduced 14 days after the first MeHg exposure (Day 14) but gradually recovered by Day 70 (Figure 1A,B). The thickness of A-fibers was significantly increased on Day 14, likely reflecting Wallerian degeneration, but rapidly returned to baseline levels thereafter (Figure 1C). In contrast, both the number and the thickness of the C-fibers remained unaffected at all time points examined (Figure 1D,E).

### 2.2. Temporal Dynamics of DRG Neurons

**Figure 2:**
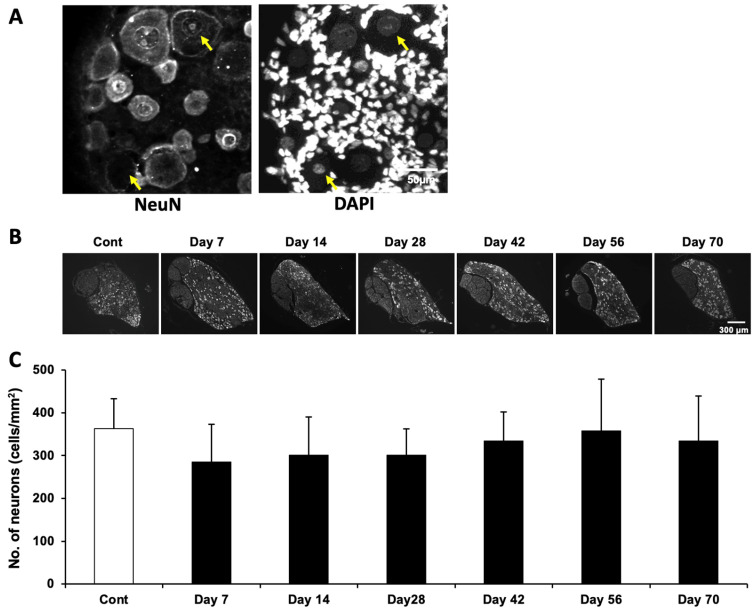
The number of morphologically identified neurons in the DRG was not affected in MeHg-exposed rats. (**A**) A representative image of the NeuN and DAPI staining of the Day 14 sample. Arrows indicate neurons that are NeuN-negative but remain morphologically intact based on their cell surface features and nuclear appearance. (**B**) Representative images of the NeuN-immunostained DRG at each time point. Note that NeuN-positive neurons look downregulated especially in the Day 14 sample. The scale bars apply to all corresponding images. (**C**) The average number of neurons that contain NeuN-positive or morphologically identified neurons. Data are shown as mean ± SD (*n* = 3). One-way ANOVA with post hoc Tukey–Kramer test. The degrees of freedom were 20 (total), 6 (between groups), and 14 (error variance) for all analyses. *p* = 0.411; F = 1.04.

We previously reported that the number of NeuN-positive neurons in the DRG was significantly decreased on Day 14 in MeHg-exposed rats [8]. However, as shown in Section 2.3, the expression of neuronal marker genes and proteins in the DRG was markedly diminished by the MeHg exposure, particularly at Day 14. Although the number of NeuN-immunopositive neurons was significantly reduced at this time point, NeuN-immunonegative neurons were still present in the DRG (Figure 2A). Furthermore, DRG neurons are widely recognized as being readily identifiable based on their substantially larger somata and nuclei compared with all other cell types present in the ganglion [26,27,28]. Therefore, we carefully identified and counted viable neurons based on their morphology, diameter, and distribution within the DRG using NeuN-immunostained samples (Figure 2B). Surprisingly, under our experimental conditions, the total number of neurons was not affected by the MeHg exposure. Moreover, although the difference did not reach statistical significance, the neuronal count appeared to increase slightly by Day 70.

### 2.3. Temporal Dynamics of Subtype Marker Genes in DRG Neurons

**Figure 3:**
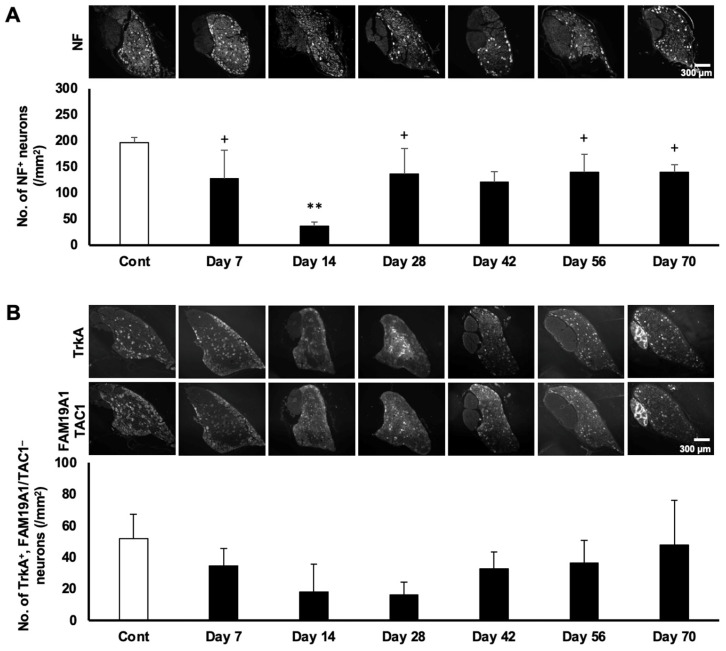

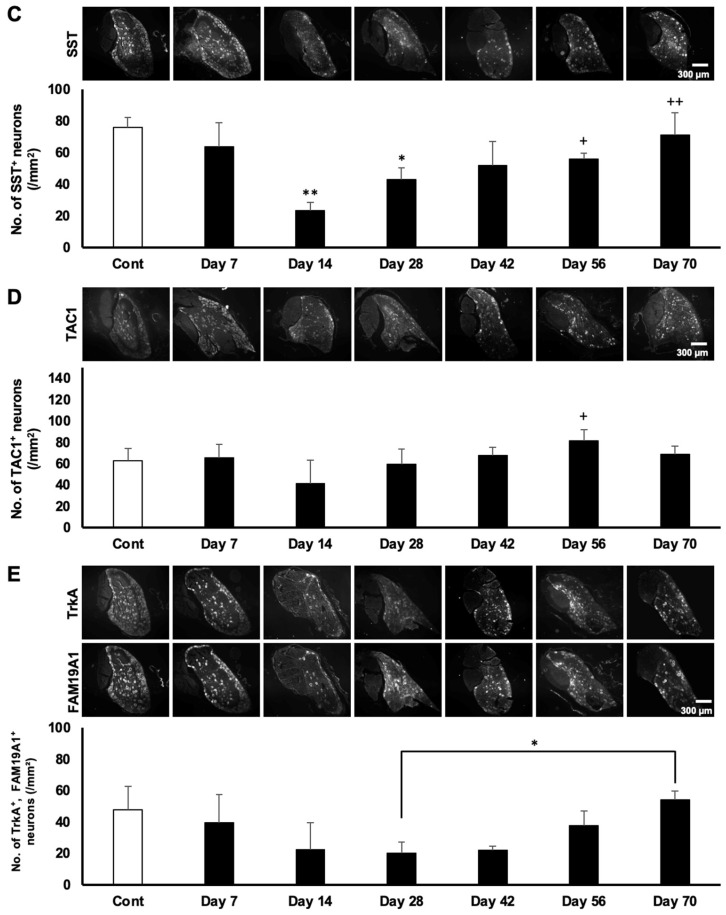
Temporal downregulation and subsequent recovery of the number of neuronal subpopulations. (**A**) Representative images of NF staining in each time point (upper) and the average number of NF-positive neurons (lower). *p* = 0.002; F = 6.46. (**B**) Representative images of TrkA and FAM19A1/TAC1 staining at each time point (upper) and the average number of TrkA-positive with FAM19A1/TAC1-negative neurons (lower). *p* = 0.125, F = 2.05. (**C**) Representative images of SST staining at each time point (upper) and the average number of SST-positive neurons (lower). *p* = 0.00059; F = 8.77. (**D**) Representative images of TAC staining at each time point (upper) and the average number of TAC-positive neurons (lower). *p* = 0.073; F = 2.51. (**E**) Representative images of TrkA and FAM19A1 staining at each time point (upper) and the average number of TrkA- and FAM19A1-double-positive neurons (lower). *p* = 0.018; F = 3.83. The scale bars apply to all corresponding images. Data are shown as mean ± SD (*n* = 3). One-way ANOVA with post hoc Tukey–Kramer test. The degrees of freedom were 20 (total), 6 (between groups), and 14 (error variance) for all analyses. The corresponding *p*-values and F-values are shown in each figure legend. * *p* < 0.05 and ** *p* < 0.01 compared with control; + *p* < 0.05 and ++ *p* < 0.01 compared with Day 14, respectively, unless otherwise specified.

**Figure 4:**
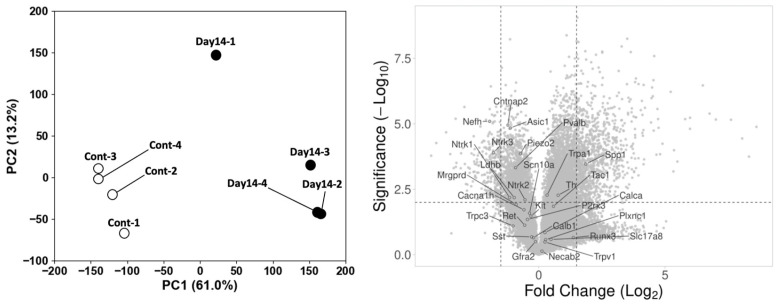
Principal component analysis (PCA) and volcano plot of the Day 14 samples compared with the control group in GSE312165. The volcano plot is annotated with genes specifically expressed in sub-neuronal populations.

DRG neurons in rodents are known to be subdivided into 11 distinct subtypes based on their gene expression profiles [17]. We previously reported that a selective attenuation of nociception (hypoalgesia) was observed in MeHg-exposed rats under the same experimental conditions [9]. To clarify whether nociceptive neurons are specifically affected by MeHg exposure, we performed immunostaining of the DRG using several subtype-specific markers to distinguish mechanoreceptive and nociceptive neurons. Mechanoreceptive neurons were identified by NF expression (Figure 3A). The number of NF-positive neurons was significantly reduced at Day 14 and gradually recovered by Day 70. Non-peptidergic nociceptive neurons were defined as TrkA-positive but FAM19A1/TAC1-double-negative or SST-positive neurons (Figure 3B,C). Peptidergic nociceptive neurons were defined as TAC1-positive or TrkA/FAM19A1-double-positive neurons (Figure 3D,E). Similarly to mechanoreceptive neurons, all nociceptive subtypes exhibited a comparable temporal pattern. In parallel, we performed a DNA microarray analysis to assess whether the transcription of neuronal subtype-specific markers was similarly affected by MeHg exposure. Consistent with the immunohistochemical findings in Figure 3, most subtype-specific marker genes were downregulated at Day 14 (Figure 4, the DNA microarray data have been deposited in the NCBI GEO database under accession number GSE312165). These results indicate that both nociceptive and mechanoreceptive DRG neurons are comparably impacted by MeHg exposure. Therefore, the previously reported selective attenuation of nociception may primarily reflect changes in the central, rather than the peripheral, nervous system.

### 2.4. Spatiotemporal Dynamics of Hg Accumulation in the DRG

**Figure 5:**
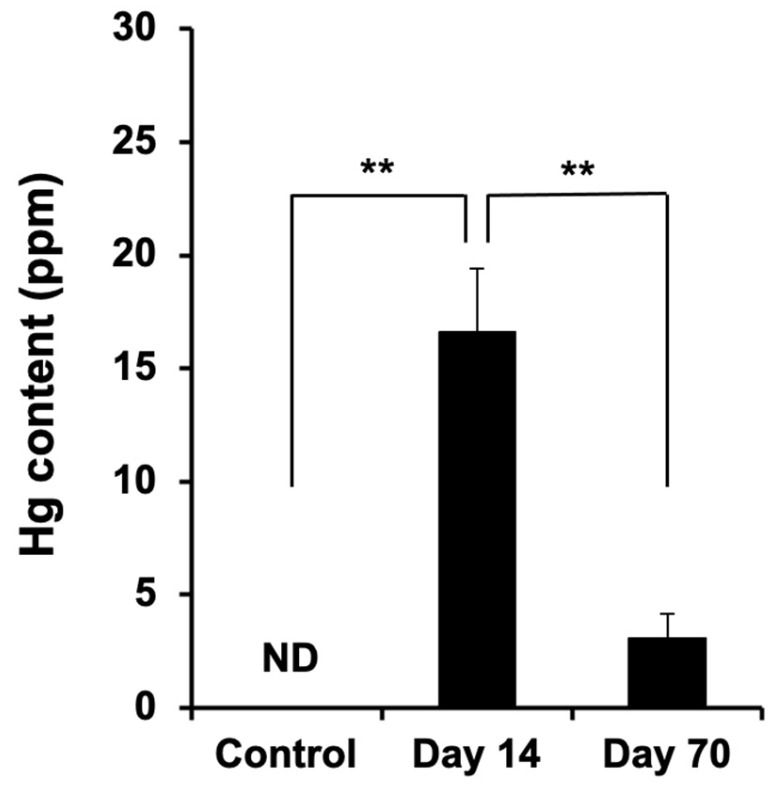
Temporal Hg contents in DRG. ND: not detected. Data are shown as mean ± SD (*n* = 3). One-way ANOVA with post hoc Tukey–Kramer test. ** *p* < 0.01. The degrees of freedom were 11 (total), 2 (between groups), and 9 (error variance) for all analyses. *p* = 5.92 × 10^−7^; F = 104.4.

**Figure 6:**
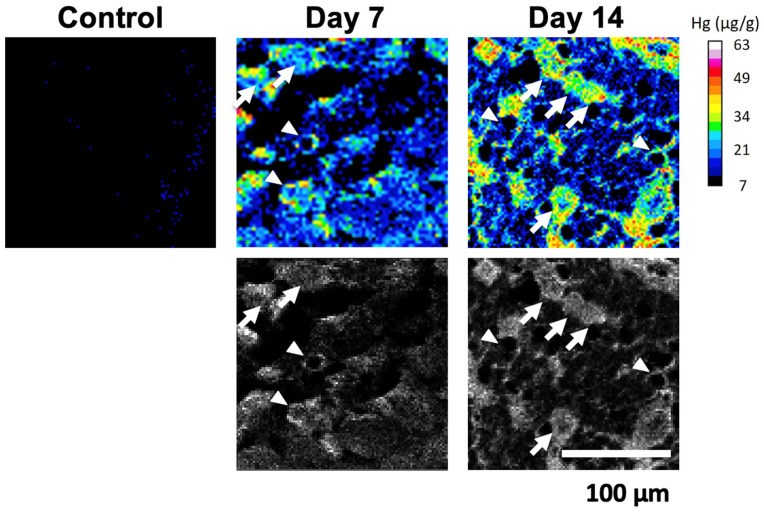
Hg distribution in DRG. Hg distributed in DRG neurons (arrows) and SGCs (arrowheads). The scale bar applies to all corresponding images.

The temporal and spatial accumulation of MeHg is presumed to influence the neuronal gene expression and function in the DRG. The total mercury in the whole DRG was significantly higher at Day 14 than in controls (Figure 5). By Day 70, total Hg levels had returned to control values. The spatial mapping of Hg in DRG sections revealed that the predominant accumulation site was the neuronal somata (Figure 6). A secondary accumulation was observed in the surrounding SGCs. Notably, the images suggest an initial buildup in SGCs followed by a gradual redistribution into neurons (see arrows and arrowheads in Figure 6 from Day 7 to 14). Collectively, these data indicate that neuronal accumulation of total mercury is associated with neuronal dysfunction, whereas the subsequent decline in total tissue mercury over time may permit functional recovery in neurons that remain viable.

## 3. Discussion

This study delineates the time course and cellular loci of MeHg-induced injury and recovery in the DRG and peripheral sensory fibers. Four principal observations emerge. First, large myelinated A-fibers underwent transient degeneration—evidenced by reduced counts and an increased fiber caliber at Day 14—followed by structural recovery by Day 70, whereas C-fibers were largely spared. Second, despite a marked reduction in NeuN immunoreactivity at Day 14, the total number of morphologically identified neurons in the DRG remained unchanged across time points. Third, both mechanoreceptive and nociceptive neuronal subtypes showed a parallel, transient reduction in subtype-specific marker expression that recovered by Day 70, a pattern corroborated at the transcript level by the DNA microarray. Fourth, the total mercury in the bulk DRG peaked at Day 14 and was normalized by Day 70, while the spatial mapping indicated predominant Hg localization to the neuronal somata with a secondary accumulation in SGCs; notably, signals suggested an early enrichment in SGCs preceding neuronal accumulation.

A central implication of our findings is that MeHg exposure under the present regimen induces a reversible phenotypic suppression of DRG neurons rather than frank neuronal loss. The apparent “loss” of NeuN-positive cells at Day 14 contrasts with the stable counts of morphologically identified neurons, suggesting that the NeuN downregulation accounts for the immunohistochemical signal drop. Indeed, the DNA microarray analysis demonstrated that the expression level of NeuN mRNA (gene symbol *Rbfox3*) was decreased by more than 50% at Day 14. This interpretation is reinforced by the coordinated, transient downregulation of subtype markers at both protein and mRNA levels, indicating a broad suppression of neuronal identity programs during the peak Hg burden. Such reversible transcriptional/antigenic silencing aligns with the recovery of the marker expression and of A-fiber structure by Day 70, when the total Hg had returned to baseline. A similar gene expression pattern has been reported, mirroring the TRPV1 profile after a perineural capsaicin treatment [29], and such transcriptional reprogramming triggered by a peripheral nerve injury is well documented [30]. Additionally, although the length of the comparison period and species differences must be considered, the 25-year longitudinal follow-up data on patients with Minamata disease—showing recovery trends in sensory symptoms in many cases—are consistent with our present findings [31]. Together, these observations argue that MeHg primarily depresses neuronal phenotypes and functions in surviving DRG neurons rather than inducing widespread cell death within the timeframe studied.

The selective vulnerability of A-fibers (i.e., large myelinated fibers that mediate mechanoreception, proprioception, and nociception), with the relative sparing of C-fibers (i.e., small unmyelinated fibers that mediate nociception and thermoreception), provides a mechanistic basis for the classical sensory phenotype of Minamata disease. These large myelinated fibers, which mediate touch and proprioception, showed a transient loss and increased caliber on Day 14, followed by a structural restitution by Day 70—findings consistent with Wallerian degeneration and the subsequent recovery. In contrast, these small unmyelinated fibers remained numerically and morphometrically stable. It seems reasonable to interpret Wallerian degeneration as being observed in the form of pathological axonal swellings. However, changes in the number of NF-labeled axons may reflect not only axonal degeneration but also a net reduction in NF-positive axons due to decreased NF expression levels. Indeed, reductions in NF expression have been indicated both in assessments of NF-positive neuronal cell numbers in the DRG and in DNA microarray analyses. Therefore, it may be premature to conclude that this solely represents a loss of A-fibers, and more detailed analyses using electron microscopy or other approaches will be required. Nevertheless, biopsies from patients with Minamata disease similarly demonstrate a degeneration of A-fibers, while C-fibers show no loss or even an increase [5,32]. Electrophysiological studies of Minamata disease patients have likewise reported similar tendencies [33,34]. Importantly, our prior behavioral work demonstrated hypoalgesia and its recovery under the same exposure conditions [9]. In other words, the present observations suggest that the hypoalgesia in the acute phase may result from the loss of A-fibers, whereas, as described later, the current histological stability of C-fiber counts therefore implies that nociceptive deficits may arise from alterations upstream in central nociceptive circuits.

The temporal alignment between the peak Hg content (Day 14), nadir of neuronal marker expression, and maximal structural perturbation of A-fibers suggests a causal link between the local Hg burden and neuronal dysfunction. Spatially, Hg signals were concentrated in the neuronal somata, with an appreciable accumulation in SGCs, and the imaging sequence suggests an initial SGC uptake followed by a redistribution to the neurons. SGCs tightly ensheathe the DRG somata and regulate the perineuronal milieu; they may act as an initial sink and subsequent source for MeHg transfer, potentially via amino acid transport systems (e.g., LAT1-mediated uptake of MeHg–Cys conjugates) and gap junctional coupling [15,35]. SGC activation can also modulate neuronal excitability through paracrine signaling [36]. Although our data do not directly measure glial reactivity, the inferred SGC–neuron transfer dynamics offer a plausible route by which MeHg achieves high neuronal somatic concentrations that transiently repress gene expression programs.

After a long period following the cessation of the MeHg administration, the total mercury levels in the DRG were significantly reduced. Numerous studies have investigated the in vivo and intracellular kinetics of MeHg, as well as its excretion mechanisms. In general, MeHg is thought to form conjugates with cysteine, selenocysteine, and glutathione [37] and to be gradually excreted in the urine [38]. On the other hand, it is also known that MeHg can undergo demethylation to inorganic mercury within the bodies of mammals [39,40]. Previous studies have shown that demethylation occurs in the liver [41] and in astrocytes in the brain [42], and although direct evidence is lacking for the DRG, a similar demethylation of MeHg may occur there as well. Once converted into its inorganic form, membrane permeability decreases [43], and in some cell types the influence of inorganic mercury may therefore be more pronounced [44,45]. In any case, under the conditions of the present study, the mercury accumulation decreased to a level that was no longer significantly different from the control group by Day 70. Furthermore, the associated histological phenotypes had recovered, at least to the extent that we were able to examine. These findings suggest that the metabolism and excretion of MeHg were functioning appropriately.

Our integrated dataset favors a model in which the peripheral DRG undergoes a reversible functional depression during peak MeHg loads, with limited permanent cytotoxicity under the present dosing paradigm. This reconciles with our earlier observation of selective hypoalgesia [9] when considering that (i) C-fiber numbers are preserved; (ii) nociceptor markers are transiently suppressed; and (iii) the total Hg declines to baseline by Day 70, concomitant with the recovery of molecular and structural readouts. Hence, persisting sensory abnormalities beyond the acute phase may reflect central mechanisms—e.g., MeHg’s effects on the brain—rather than sustained peripheral neuron loss. In contrast to the transient hypoalgesia, the hind limb crossing score remained elevated up to Day 70, suggesting that the central nervous system had sustained a permanent (at least during the course of our experimental period) impairment [9]. Testing this will require a coordinated assessment of the central nuclei alongside the DRG over matched time courses.

Several caveats temper our conclusions. First, NeuN-negative but morphologically defined neurons likely include small-diameter populations, while our approach mitigates antigen-loss bias, morphology-based identification can undercount the smallest cells or inflate counts under edema. Second, our microarray analysis captures population-level transcriptional changes; single-cell resolution would more definitively ascribe dynamics to specific subtypes and identify rare, vulnerable populations. Third, the Hg measurements reflect the total Hg and do not distinguish MeHg from demethylated inorganic Hg and/or other Hg-containing molecules; speciation would sharpen the mechanistic inference. Finally, the sample size (*n* = 3 per time point for several assays) limits the power for detecting subtle effects, especially at Day 70 where trends toward increased neuronal counts did not reach significance.

Building on these findings, several lines of inquiry appear most informative. (i) Cell-state resolution: single-nucleus RNA-seq or spatial transcriptomics across Days 0–70 to chart subtype-specific transcriptional trajectories and glial responses. (ii) Function: in vivo electrophysiology or calcium imaging of DRG neurons to correlate marker suppression with excitability and stimulus coding, complemented by quantitative sensory testing. (iii) Mechanism: the pharmacological or genetic manipulation of candidate uptake pathways (e.g., LAT1) and SGC–neuron coupling (e.g., connexins) to test their roles in MeHg distributions and neuronal silencing. (iv) Speciation and flux: longitudinal MeHg/inorganic Hg speciation in the DRG and central sensory relays to map detoxification and redistribution kinetics. (v) Translation: the definition of exposure thresholds that separate reversible suppression from irreversible loss may inform clinical monitoring and intervention windows in MeHg intoxication.

## 4. Materials and Methods

### 4.1. Animals, MeHg Administration, and Study Design

All experimental procedures were conducted in compliance with the Regulations for Animal Research at Tokyo University of Pharmacy and Life Sciences, with prior approval obtained from the institutional ethics committee. Efforts were undertaken to minimize the number of animals used as well as their discomfort and distress. Eight-week-old male Wistar rats (Japan SLC Inc., Hamamatsu, Japan) were purchased and maintained in standard housing conditions under a 12 h light/dark cycle, with food and water provided ad libitum. From the following week (9 weeks old), MeHg (Merck Millipore, Burlington, MA, USA) was administered following previously established protocols [46]. A MeHg solution (2 mg/mL in water) was administered orally via gastric intubation at a daily dose of 6.7 mg/kg for five consecutive days, followed by a two-day treatment-free period. This regimen was repeated once. Age-matched control rats were administered an equivalent volume of water per kilogram of body weight. A total of 38 rats were used in this study (21 for immunohistochemistry and Hg distribution, 8 for DNA microarray analysis, and 9 for mercury content measurement). Body weights ranged from approximately 200 to 300 g (the control group was around 300 g, and the MeHg-exposed group was around 200 g at the time of dissection).

This study was designed as an in vivo toxicological experiment to investigate the temporal effects of methylmercury exposure on neuronal cells and nerve fibers in the rat DRG. Rats were exposed to MeHg for a defined period, and DRG tissues were collected at predetermined time points before and after exposure to assess time-dependent morphological changes.

### 4.2. Immunohistochemistry

Immunohistochemistry was performed as described previously with minor modification [47]. Briefly, deeply anesthetized rats were transcardially perfused with PBS followed by 4% paraformaldehyde (PFA; FUJIFILM Wako Pure Chemical, Osaka, Japan) in 0.1 M phosphate buffer. DRGs together with sensory nerves were dissected from the lumbar region (L4–6). Tissues were postfixed overnight at 4 °C in 4% PFA/0.1 M PB then cryoprotected in 20% sucrose in PBS overnight at 4 °C. Samples were embedded in Tissue-Tek OCT compound (Sakura Finetek, Tokyo, Japan), frozen at −78 °C, and sectioned at 14 µm thickness using a cryostat (HM550; Thermo Fisher Scientific, Tokyo, Japan) at −20 °C. Sections were air dried overnight at 37 °C and stored at −80 °C until use.

For immunohistochemistry, sections were washed in PBS and blocked for 30 min in antibody buffer (2× PBS containing 2% BSA, 0.1% Triton X-100, and 0.05% NaN_3_). Samples were incubated with primary antibodies either for 1 h at room temperature or overnight at 4 °C, followed by PBS washes and incubation with secondary antibodies for 1 h at room temperature. Stained sections were mounted with Fluoromount/Plus containing DAPI (Roche Diagnostics, Mannheim, Germany) and imaged using a fluorescence microscope (TS-100; Nikon, Tokyo, Japan) equipped with a CMOS camera (Zyla 5.5; ANDOR, Tokyo, Japan). Image acquisition and processing were performed with NIS-Elements software (Version 4.50; Nikon) and Photoshop (Version 23.3.1; Adobe, San Jose, CA, USA), and quantitative analyses were conducted using Photoshop for visual inspection and ImageJ software (Version 1.52k; NIH, Bethesda, MD, USA) for digital quantification.

Primary antibodies included NeuN (Merck Millipore; ABN91, 1:1000), neurofilament (NF; Merck Millipore; N2912, 1:1000), TrkA (R&D Systems, Minneapolis, MN, USA; AF1056, 1:1000), FAM19A1 (Merck Millipore; HPA013407, 1:1000), TAC1 (Immunostar, Hudson, WI, USA; 20064, 1:1000), and SST (Abcam, Tokyo, Japan; ab111912, 1:1000). Secondary antibodies were Alexa Fluor 488-, 555-, or 647-conjugated goat anti-mouse, anti-rabbit IgG, or anti-chicken IgY (Thermo Fisher Scientific; A11029, A21422, A21235, A32731, A21429, A21245, A21437, A21449, respectively, 1:2000).

### 4.3. DNA Microarray and Bioinformatics

RNA amplification, labeling, and hybridization were performed as previously described with minor modifications [48]. Control and Day 14 rats were deeply anesthetized with CO_2_, and DRGs were rapidly dissected from the lumbar region. Samples were frozen at −80 °C until analysis. Total RNA was amplified and labeled with Cyanine-3 (Cy3) using the Low-Input Quick Amp Labeling Kit, One-Color (Agilent Technologies, Santa Clara, CA, USA) according to the manufacturer’s instructions. Briefly, total RNA was reverse-transcribed into double-stranded cDNA using a poly (dT)–T7 promoter primer. The primer, template RNA, and quality control transcripts of known concentration and quality were denatured at 65 °C for 10 min and then incubated at 40 °C for 2 h in 5× First Strand Buffer containing 0.1 M DTT, 10 mM dNTP mix, and AffinityScript RNase Block Mix. The AffinityScript enzyme was inactivated at 70 °C for 15 min.

The resulting cDNA was used as a template for in vitro transcription to generate fluorescent cRNA. cDNA was combined with a transcription master mix containing T7 RNA polymerase and Cy3-labeled CTP and incubated at 40 °C for 2 h. Labeled cRNA was purified using RNeasy Mini spin columns (Qiagen, Venlo, The Netherlands) and eluted in 30 µL of nuclease-free water. After amplification and labeling, cRNA yield and Cy3 incorporation were assessed with a NanoDrop ND-1000 spectrophotometer (ThermoFisher Scientific) and an Agilent Bioanalyzer (Agilent Technologies). For each hybridization, 0.60 µg of Cy3-labeled cRNA was fragmented and hybridized to an Agilent SurePrint G3 Rat Gene Expression v2 8×60K Microarray (Design ID 074036) at 65 °C for 17 h. After washing, arrays were scanned using an Agilent DNA microarray scanner.

DNA microarray analysis was performed as previously described [49,50,51]. Intensity values for each scanned feature were quantified with Agilent Feature Extraction (v11.5.1.1) with background subtraction enabled. Only features flagged as detected (no errors) were retained; features flagged as not detected or compromised—including not positive and significant, non-uniform, not above background, saturated, or population outliers—were excluded. Normalization was performed in Agilent GeneSpring (v14.8) using per-chip 75th-percentile shift normalization. The Agilent SurePrint G3 Rat Gene Expression v2 8×60K Microarray (Design ID 074036) contains 45,598 non-control probes. Four biological replicates per group (control and Day 14; 8 samples total) were analyzed. Differential expression was assessed by one-way ANOVA followed by Tukey’s honestly significant difference (HSD) post hoc test, with multiple testing controlled by the Benjamini–Hochberg false discovery rate (FDR). Principal component analysis (PCA) and visualization were executed using Python (Version 3.14.1), and Volcano plot construction was carried out with Python and visualized by VolcaNoseR website (https://huygens.science.uva.nl/VolcaNoseR/ (accessed on 4 December 2025)).

### 4.4. Determination of Total Mercury in DRG

Mercury measurements were performed as previously described [52]. Rats were deeply anesthetized with CO_2_, and DRGs were rapidly dissected from the lumbar region. Samples were frozen at −80 °C until analysis. Total mercury in DRGs was quantified using a direct mercury analyzer (MA-3000; Nippon Instruments Corporation, Kyoto, Japan). Approximately 5 mg (wet weight) of each sample was weighed on an analytical balance, loaded directly onto a ceramic boat, and analyzed in solid sample mode. Concentrations were determined by external calibration with mercury standard solutions and expressed relative to tissue wet weight.

### 4.5. Spatiotemporal Distribution of Hg in DRG

Fourteen-micrometer DRG slices, prepared using the same procedure as for immunohistochemistry, were mounted on a six-micrometer-thick polypropylene film (Rigaku Holdings Corporation, Tokyo, Japan) and air dried in a clean box at room temperature for 1 h. The specimens were stored in a desiccator until analysis. Mercury distribution in DRG was analyzed by synchrotron radiation X-ray fluorescence (SR-XRF) analysis with microprobe. SR-XRF measurements were performed at BL37XU of SPring-8 (Sayo, Japan) using a scanning X-ray micro-spectroscopy system with 30 keV monochromatic X-rays (beam size, 1 μm × 1 μm) [53]. The resolution of the obtained maps was 1 μm/step. The X-ray fluorescence of the Hg Lα line was measured at each point using a Ge solid-state detector (EGX10-06-CP5-PLUS-WC; Mirion Technologies, Atlanta, GA, USA). While scanning, the specimens were processed using a personal computer, and the elemental maps are shown in 8-bit grayscale from the lower detection limit up to the maximum in linear proportion to the element concentration. Hg in microregions was quantified using thin section standards of Hg for microbeam analysis (10 µm; 0–100 µg/g) [54]. In brief, the calibration line was obtained from the mean value of the total X-ray intensity of Hg Lα line in 1 × 1 μm^2^ area of the 25 measurement points in each section standard, and thickness compensation was applied according to the sample thickness (14 µm).

### 4.6. Statistical Analysis

All statistical analyses except for microarray data were performed using Excel (Microsoft, Redmond, WA, USA) with the Statcel5 add-in software (OMS, Tokyo, Japan). Data are presented as the mean ± SD. Differences among multiple groups were evaluated using one-way ANOVA followed by the post hoc Tukey–Kramer test. All data collection and analyses were conducted in a double-blind manner.

## 5. Conclusions

In summary, MeHg exposure in rats induces a transient, Hg burden-linked suppression of the DRG neuronal phenotype with a selective structural vulnerability of A-fibers and a relative preservation of C-fibers. Neuronal counts remain stable, the subtype marker expression and A-fiber structure recover as the total Hg normalizes, and Hg localizes primarily to the neuronal somata with a contributory accumulation in SGCs. These features support a model of reversible peripheral neuronal dysfunction rather than pervasive neuron loss, and they raise the possibility that sustained nociceptive deficits after MeHg exposure may be driven, at least in part, by central mechanisms.

## Data Availability

The original contributions presented in this study are included in the article. Further inquiries can be directed to the corresponding authors.
